# Academic burnout in the TikTok era: a battle between pleasure and concentration

**DOI:** 10.3389/fpsyg.2026.1774030

**Published:** 2026-03-05

**Authors:** Yanning Chen, Jimin Hu, Li Cheng

**Affiliations:** 1 School of Languages and Cultures, Hunan Institute of Technology, Hengyang, Hunan, China; 2 Department of Foreign Languages, Ganzhou Teachers College, Ganzhou, Jiangxi, China

**Keywords:** declined concentration, English-learning burnout, short videos, the self-determination theory, TikTok brain

## Abstract

**Introduction:**

The negative connection between short-video excessive usage habits and students’ learning is becoming increasingly prominent in the field of education. With the excessive use of short videos, a phenomenon known as the “TikTok brain” has emerged. However, the research on the “TikTok brain” variable and its understanding are still at the initial exploration stage. This study tries to propose seven research hypotheses and build a corresponding theoretical model based on the self-determination theory, attempting to explore the relationship and transmission path between the TikTok brain, declined attention, and learning burnout.

**Research methods:**

students are both the main users of short videos and the high-risk group for learning burnout. Therefore, this study collected 500 valid questionnaires to verify the above research hypotheses. Among them, there are 243 male students (48.6%) and 257 female students (51.4%).

**Results:**

(1) There is a significant positive correlation between the TikTok brain and declined attention; (2) The TikTok brain is also significantly positively correlated with learning burnout; (3) Declined attention plays an effective mediating role between the TikTok brain and learning burnout. This study concludes that the immediate satisfaction cognitive model formed by short-video excessive usage will further intensify students’ learning burnout through consuming their attention resources. This also provides an insight into educational practice. In the educational scenario, paying attention to guiding and intervening in students’ digital usage habits to help them cultivate and maintain a sustainable learning state is necessary.

## 1 Introduction

With their short, flat, quick content features and exact algorithmic recommendations, short-video apps like TikTok have transformed the information acquisition and entertainment habits of hundreds of millions of people in the present age, when digital media are thoroughly incorporated into daily life (Ye et al., 2025). They have also sparked a cool culture based on immediate gratification. Through very compressed stories, strong emotional stimulation, and continually decreasing interaction designs, this culture lets users rapidly enter the flow state at very little cognitive cost and have high-frequency pleasure experiences (Su et al., 2021). This extremely stimulating and segmented pleasure pattern could have a significant effect on consumers’ cognitive abilities—particularly their self-regulation and focus. Over the past three years, neuroscience and psychological studies have consistently provided updated evidence for this, showing that excessive short videos viewing is strongly linked with reduced prefrontal lobe activity, fragmented attention, and reduced self-control (Ye et al., 2024; Brunborg and Andreas, 2019). Further neuroscientific results demonstrate that repeated exposure to short videos recruits dopaminergic brain regions (e.g., substantia nigra), leading to a real or perceived pleasure improvement, and is associated with phenomena labeled as the “TikTok brain” (Ye et al., 2025; Su et al., 2021). TikTok brain (perceived pleasure enhancement) refers to the psychological dependence state formed by individuals after long-term excessive exposure to short videos. It is specifically manifested as a high degree of reliance on the stimulation provided by short videos to bring about a sense of pleasure (Su et al., 2021). This psychological dependence state needs the sustained dopamine release to reinforce users’ appetite for quick reward cycles and can have negative consequences in educational settings, where it might foster impatience and inability to focus (Li et al., 2021). However, the research on the impact of this highly dependent psychological state and its negative effects on students’ use of new media is still insufficient. Ye et al. (2024) emphasized that people need to have a clearer understanding of the obvious negative psychological and neurological effects brought about by the TikTok brain. Therefore, this study regards the TikTok brain as the main variable to carry out a more in-depth investigation.

Meanwhile, in the field of higher education, an increasing number of college students are showing obvious signs of burnout during the process of learning English, including emotional exhaustion, decreased learning efficiency, and even a sense of estrangement from knowledge itself (Huang, 2025). This phenomenon is usually due to the heavy schoolwork, lack of motivation, or problems with teaching methods. However, it is worth noting that, as watching short videos has become the mainstream way for young people to relax and obtain information in their daily lives, this content consumption habit may be quietly changing our cognitive patterns and thus giving rise to a new type of learning burnout (Mao and Liao, 2025). Learning English itself requires continuous concentration, in-depth thinking, and long-term accumulation, while short videos offer immediate satisfaction and the experience of quick switching (Yu et al., 2022). When the brain has adapted to high-frequency stimulation and fragmented information reception methods, it is inevitable to experience cognitive discomfort and psychological resistance when one needs to calm down to face deep learning, which requires patience and perseverance (Pham et al., 2025). This internal conflict might precisely be the key reason why many young people find it particularly difficult to study and are more prone to burnout. Currently, the research on academic burnout is gradually gaining attention in the field of English as a Foreign Language (EFL). Existing studies mostly focus on students in majors such as nursing and medicine (Hwang and Kim, 2022; Dunn et al., 2008), or concentrate on the causes and consequences of burnout among English teachers (Pishghadam et al., 2021; Saboori and Pishghadam, 2016). In contrast, discussions on burnout among English learners themselves are still relatively limited. A few studies have shown that both English majors and non-majors generally experience moderate levels of burnout (Yu et al., 2022; Wang et al., 2018). However, existing research has not yet verified the relationship between pleasure enhancement and English-learning burnout among Chinese undergraduates in the era of short videos, as well as the mediating role of the decline in concentration. Filling these gaps in the literature is the purpose of this research.

## 2 Literature review and hypotheses

### 2.1 The self-determination theory (SDT)

**FIGURE 1:**
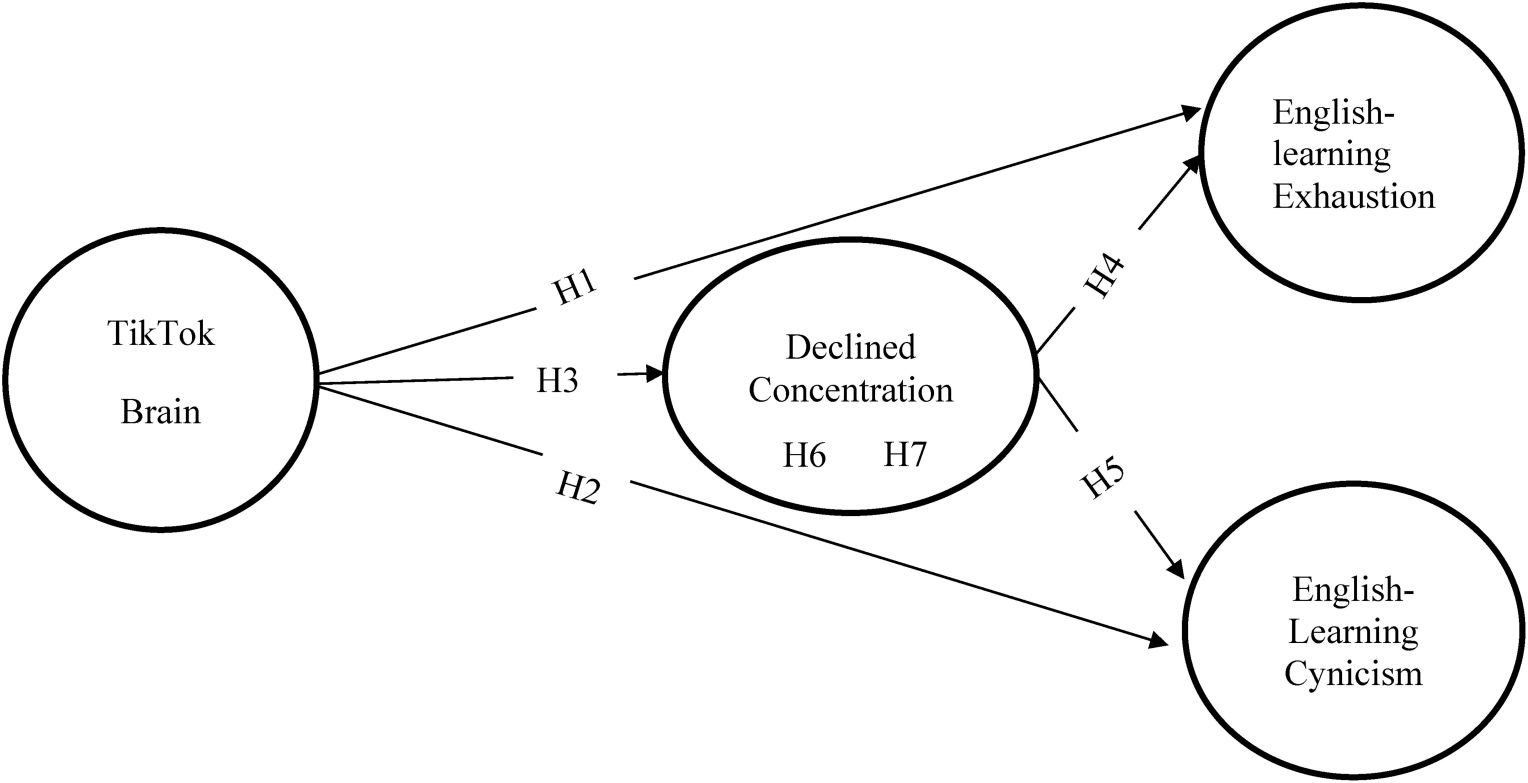
Proposed research model.

With digital media profoundly transforming the learning environment now, this study applies the self-determination theory (SDT) as its main structure to thoroughly investigate the internal psychological mechanism between students’ learning burnout and the perceived pleasure from watching short videos on TikTok. According to SDT, autonomy, competence, and relatedness are the three fundamental psychological needs that an individual’s persistent positive behavior and intrinsic motivation come from Deci and Ryan (2012). As a sophisticated cognitive activity, English learning’s effective execution and continuous investment depend precisely on the following aspects: students must experience independent control over the learning process, develop genuine language ability improvement by overcoming challenges, and create an academic sense of belonging through interactions with teachers, peers, and classmates (Chiu, 2024; Guay, 2022). TikTok platform can quickly meet users’ superficial pleasure needs by providing immersive experiences and immediate interactive feedback (Ye et al., 2022). However, this easily accessible pseudo-autonomy and pseudo-competence will occupy limited psychological resources, thereby weakening the true autonomy and competence needed by students when facing tasks that require long-term effort and delayed rewards (Huang et al., 2021; Huang, 2025). When these basic psychological needs are continuously frustrated, it is likely to trigger emotional exhaustion, cynicism, and low self-efficacy, which are the core manifestations of learning burnout. Therefore, this study presents the following research model (see Figure 1).

### 2.2 Research hypotheses

#### 2.2.1 The direct relationships

Chinese university students are vulnerable to burnout due to the reason that university or career choices are determined by highly competitive exams (Hu and Schaufeli, 2009). They participate in various mandatory and organized learning activities that, from a psychological perspective, may be regarded as their work. These activities include attending classes, completing tasks, and finishing exams (Salanova et al., 2010). Salanova et al. (2010) emphasized that the pain experienced by students in these exercises manifested as more general anxiety or depression, or more specific academic burnout. Academic burnout is defined by Zhang et al. (2007) as students feeling tired owing to the demands of their studies (exhaustion), feeling distant from their peers, classmates, or teachers (cynicism), and feeling inadequate or like they haven’t accomplished much as a student (inefficacy). This study agrees with Vizoso et al. (2019) that the core of burnout lies in emotional exhaustion and cynicism, as there is a powerful linkage between the two, while inefficiency has little relation with them. Therefore, we take exhaustion and cynicism as the dependent variables in this study.

TikTok brain (perceived pleasure enhancement) is further defined by Ye et al. (2025) as a psychological or cognitive behavior that develops from prolonged watching short-form videos, especially on popular short-form video apps like TikTok. A relatively high perceived level of emotional improvement indicates that individuals experience a higher level of pleasure and satisfaction when watching short videos (Calabro et al., 2022). It may associate with English learning burnout through two pathways:

Firstly, TikTok brain makes the user’s emotional system accustom to being rapidly activated by frequent and low-cost stimuli, which may lower their tolerance for the inevitable setbacks and delayed gratification encountered in the process of learning English, which predicts that they may feel particularly strained and mentally exhausted when facing English learning tasks (Huang, 2025) This study agrees with Calabro et al. (2022) that perceived pleasure from short-video addiction is positively related to learning exhaustion. English-learning exhaustion (ELE) is defined by previous scholars as a feeling of fatigue caused by the exhaustion of an emotional accumulation during the English learning process (Vizoso et al., 2019). Hence, we hypothesize:

H1: TikTok brain (TB) is positively related to English-learning exhaustion (ELE).

Secondly, TikTok brain may correlate positively with students’ cynical attitude toward learning English. English-learning cynicism (ELC) is defined by Lee et al. (2020) as an indifferent, isolated, and contemptuous attitude toward the importance of learning English. The simple and straightforward narrative logic of short videos may shape users’ expectations of quick success, devaluing the value of learning outcomes that require continuous effort. This cognitive bias may trigger indifference, estrangement, and contempt toward the significance of English learning (Li, 2024; Liao, 2024). The following hypothesis is thus formulated:

H2: TikTok brain (TB) is positively related to English-learning cynicism (ELC).

The current study agrees with earlier findings that the frequency of short video use is linked with a decrease in users’ attention positively (Xie et al., 2021; Liu et al., 2023; Brooks, 2015). Chen et al. (2023) claimed that TikTok brain is regarded as more serious than short video addiction because it defines the negative psychological and behavioral characteristics of excessive use of short videos. Additionally, this variable supports Li G. et al. (2024)’s conclusion that users with a TikTok brain are addicted to short videos. Previous studies have shown that addicted users struggle to concentrate and exhibit greater attention deficits when watching short videos (Yan et al., 2024; Ye et al., 2025). As one’s addiction to short videos increases, the ability to control concentration often declines. Furthermore, due to the design features of these platforms, compared with other types of internet addiction behaviors, addiction to short videos is more likely to result in a decline in cognitive abilities (Tian et al., 2023). Consequently, the user’s concentration control ability deteriorates with the severity of overwatching short videos. Declined concentration is proposed by Ye et al. (2025), which is used to describe how prolonged, excessive exposure to short videos deteriorate a person’s ability to manage and allocate attention resources. Therefore, the following hypothesis is formulated:

H3: TikTok brain (TB) is positively related to the declined concentration (DC).

Students with poor concentration have to exert more mental effort to counteract their ineffective information processing in order to achieve learning goals, such as understanding challenging English texts, thereby accelerating the consumption of their emotional and cognitive resources. Maslach et al. (2001) suggested that when people feel that their resources (such as concentration and mental energy) are constantly depleted and cannot be fully replenished, they will experience severe emotional exhaustion. Direct evidence from empirical research also supports this path; for instance, Kirschner and De Bruyckere (2017) confirmed that distractions and sustained partial attention influenced by digital media increase students’ cognitive burden and are directly related to higher academic pressure and fatigue. Therefore, forcing people to exceed their capabilities with limited means, it predicts the development of learning exhaustion. Therefore, we put forward the following hypothesis:

H4: Declined concentration (DC) is positively related to English-learning exhaustion (ELE).

In the self-determination theory (Deci and Ryan, 2012), cynicism is manifested as questioning the value of education, cold behavior, indifference, and even satire, which is closely related to the frustration of the need for ability and autonomy (Zhang et al., 2007). This sense of frustration will be exacerbated in two aspects due to the decline in attention. First, it will immediately reduce learning efficiency. When students are unable to concentrate, they find it difficult to study deeply and understand the intrinsic value and meaning in English texts, and their learning activities become superficial and fragmented. Due to this inefficient learning method, they find it difficult to acquire a genuine sense of competence in mastering knowledge and skills (Losyeva et al., 2021). If students keep feeling frustrated, they may start to doubt the value of their learning tasks and their own talents, and thus develop negative thoughts such as “This is meaningless” or “I’m not good at this” (Li C. et al., 2024), which is cynicism. Therefore, we put forward the following hypothesis:

H5: Declined concentration (DC) is positively related to English-learning cynicism (ELC).

#### 2.2.2 The mediating relationships

Peng (2025) emphasizes that the pleasure individuals obtain from watching short videos is not free but at the cost of brain remodeling and damage to their own attention control systems. Besides, Fang et al. (2025) found that when students were unable to effectively complete their learning tasks due to addiction to short videos, it was often attribute to impaired attention. Therefore, this study suggests that TikTok brain will have a positive influence on students’ English-learning exhaustion indirectly through decreased concentration. Students will find the learning very challenging and ineffective if their attention is already influenced by short videos, and they have trouble focusing when they encounter English study demanding extended periods of concentration. Their mental energy will be quickly depleted by this never-ending process of very little outcome, leaving them very exhausted and drained. Thus, we put forward the following hypothesis:

H6: Declined concentration (DC) mediates the relationship between TikTok brain (TB) and English-learning exhaustion (ELE).

Prior study of Ye et al. (2025) confirmed that TikTok brain was associated with students’ decreased concentration positively. And decreased concentration could impact students’ learning burnout (Shah and Saleem, 2015; Polderman et al., 2010). When students are unable to concentrate, they find it difficult to complete English intensive reading, remember complex English grammar, etc. Engagement of learning is increasingly shallow and compartmentalized. This inefficient way of learning makes it difficult for them to gain a true sense of competence in the process of mastering knowledge and skills (Losyeva et al., 2021). Since declined concentration is a driver of learning cynicism, it may be an effective mediator. However, at present, the mediating role of declined concentration between the TikTok brain and learning cynicism has been overlooked. Therefore, to fill this gap, we propose the following hypotheses:

H7: Declined concentration (DC) mediates the relationship between TikTok brain (TB) and English-learning cynicism (ELC).

## 3 Methodology

### 3.1 Procedure

The Academic Committee of the Hunan Institute of Technology gave its approval to this study. The data was gathered through Wenjuanxing platform. The survey was administered anonymously using snowball sampling to Chinese full-time university students who had been using short-form video apps for a long time in August 2025. This group was chosen because it is believed that students who have used short videos more often are better able to reflect the typical consumption patterns of this group.

A statement of informed consent outlining the purpose, background, research goals, data use, and methodology of the study was presented at the start of the online survey. It also promised to protect the participants’ privacy and anonymity and included the primary investigator’s contact details. After reading the consent form and giving their informed consent, participants could choose an option. “I have understood the above material and agree to take part in this study” is the next question on the official survey. In addition, the screening question is “Have you completed at least one semester of English courses?” Only students who select “Yes” can answer all the questions. We collected 559 questionnaires. After quality inspection, we removed the invalid ones and finally had 500 valid questionnaires left. Descriptive analysis, validity analysis, reliability analysis, common method bias test, and correlation analysis were performed on TB, DC, ELE, and ELC using SPSS 27. Specifically, this study used Harman’s Single Factor test to evaluate and account for CMV. According to a principal component analysis using SPSS 27, the first factor only explained 31.124% of the total variance, which is much less than the 50% threshold (Supplementary Appendix B), indicating that CMV is not an issue (Podsakoff et al., 2003). Further, a structural equation model (SEM) was created in smart PLS 3.3.9 to test the hypotheses.

### 3.2 Participants

This study involved Chinese students who regularly use short video apps and have completed at least one semester of English. Table 1 contains information such as gender, academic year, average weekly time of use of short video applications, and average daily time of viewing short videos of participants.

**TABLE 1:** Respondents’ descriptive information (*n* = 500).

Variables	Details	Percentage (%)
Gender	Male: 243	48.6%
	Female: 257	51.4%
Subject category	The humanities: 216	43.2%
	Natural science: 218	43.6%
	Military: 66	13.2%
Academic years	Freshmen: 143	28.6%
	Sophomore: 123	24.6%
	Junior: 114	22.8%
	Seniors: 120	24%
Average days of using short-form videos platform per week	1–3: 90	18%
	4–6: 148	29.6%
	Every day: 262	52.4%
The average daily time spent watching short videos	1–2 h: 121	24.2%
	2–3 h: 158	31.6%
	3–4 h: 106	21.2%
	> 4 h: 115	23%

### 3.3 Measurements

To collect the data, a questionnaire was used. The measured variables in the questionnaire were based on earlier research. Three professors reviewed the questionnaire, and some of the procedures were changed to better suit the short video texts. Furthermore, a five-point Likert scale was applied to score the design elements, with ratings ranging from 1 to 5, meaning strongly disagree to strongly agree (Joshi et al., 2015). Since the data was gathered in China, the survey was administered in both Chinese and English. To guarantee the correctness of the translation, the study used back translation technique, which Werner and Campbell (1970) demonstrated could guarantee that the questions in the target language version were exactly the same as those in the original questionnaire. Throughout the actual execution process, the research team assigned a professor and a PhD candidate with experience in related fields who are fluent in both Chinese and English to carefully translate each question. The questions are included in the Supplementary Appendix A.

#### 3.3.1 TikTok brain

This study adopted 9 measurement items from Ye et al. (2025) to assess perceived pleasure enhancement in participants’ brain states during short-video consumption. Typical items include: “I often need to watch short videos to make me feel happier.” The scale score is positively correlated with the severity of the TikTok brain phenomenon. The higher the score, the more pronounced the related cognitive characteristics. After reliability and validity tests, Cronbach’s α was 0.933, CR was 0.944, AVE was 0.651, and FL ranged from 0.793 to 0.824, all of which met the recommended standards (Hair et al., 2019).

#### 3.3.2 Declined concentration

The measurements of Ye et al. (2025) were used in this study. This six-item assessment tool was designed specifically to gauge a participant’s ability to regulate their attention. One of the common measurement items is “I frequently have trouble concentrating on other tasks after using short video apps.” The score increases with the degree of attention control impairment. After validity and reliability tests, the Cronbach’s α was 0.892, the AVE was 0.649, the CR was 0.917, and the FL was from 0.785 to 0.823, all of which met the requirements (Hair et al., 2019).

#### 3.3.3 English-learning exhaustion

We selected the measurement items developed by Huang (2025). The original assessment was carefully reviewed and precisely modified to account for the unique learning conditions where university students experience emotional exhaustion when learning English. Only four items are covered in this assessment, such as “I feel emotionally drained by my English studies.” The severity of their learning exhaustion increases with the score. After reliability and validity testing, the Cronbach’s α, AVE, CR, and FL all met the acceptable criteria (Hair et al., 2019). The corresponding values were 0.895, 0.761, 0.927, and (0.860–0.884).

#### 3.3.4 English-learning cynicism

The measurement scale is adopted from Huang (2025). There are three items to measure ELC, for example, “I’ve become more cynical about the potential usefulness of my English studies.” The higher the score, the more severe their cynicism. After reliability and validity tests, the Cronbach’s α was 0.835, the AVE was 0.752, the CR was 0.901, and FL was (0.834–0.890), all of which met the recommended standards (Hair et al., 2019).

## 4 Results

### 4.1 Preliminary analyses

Both factor loading (FL) and average variance extracted (AVE) showed that the convergent validity meets the requirement. Besides, Table 2 showed strong discriminant validity since the square roots of the AVE were greater than the correlation coefficient between the variables that were related to them (Hair et al., 2019).

**TABLE 2:** Discriminant validity.

Variables	DC	ELC	ELE	TB
DC	0.805			
ELC	0.226	0.867		
ELE	0.226	0.545	0.872	
TB	0.212	0.181	0.155	0.807

DC, declined concentration; ELC, English-learning cynicism; ELE, English-learning exhaustion; TB, TikTok brain. The diagonal line’s value is the square root of AVE.

### 4.2 Goodness of fit assessment

Hair and Alamer (2022) suggested that the SRMR value should be between 0.08 and 0.10 to achieve an acceptable model fit. When applying CB-SEM, an SRMR value of less than 0.08 is generally considered a good model fit, but for PLS-SEM, this standard may be too low (Hu and Bentler, 1998). PLS-SEM uses a more conservative standard, so a value of less than 0.10 is considered sufficient for good model fit (Hu and Bentler, 1998). Because PLS-SEM was employed in this study, the SRMR was 0.091, which is below 0.10, meeting the model fit requirements (see Table 3).

**TABLE 3:** Model fit.

Fit indices	Saturated model	Estimated model
SRMR	0.045	0.091
Chi-square	787.844	932.055
NFI	0.886	0.865

### 4.3 Structural equation model

**FIGURE 2:**
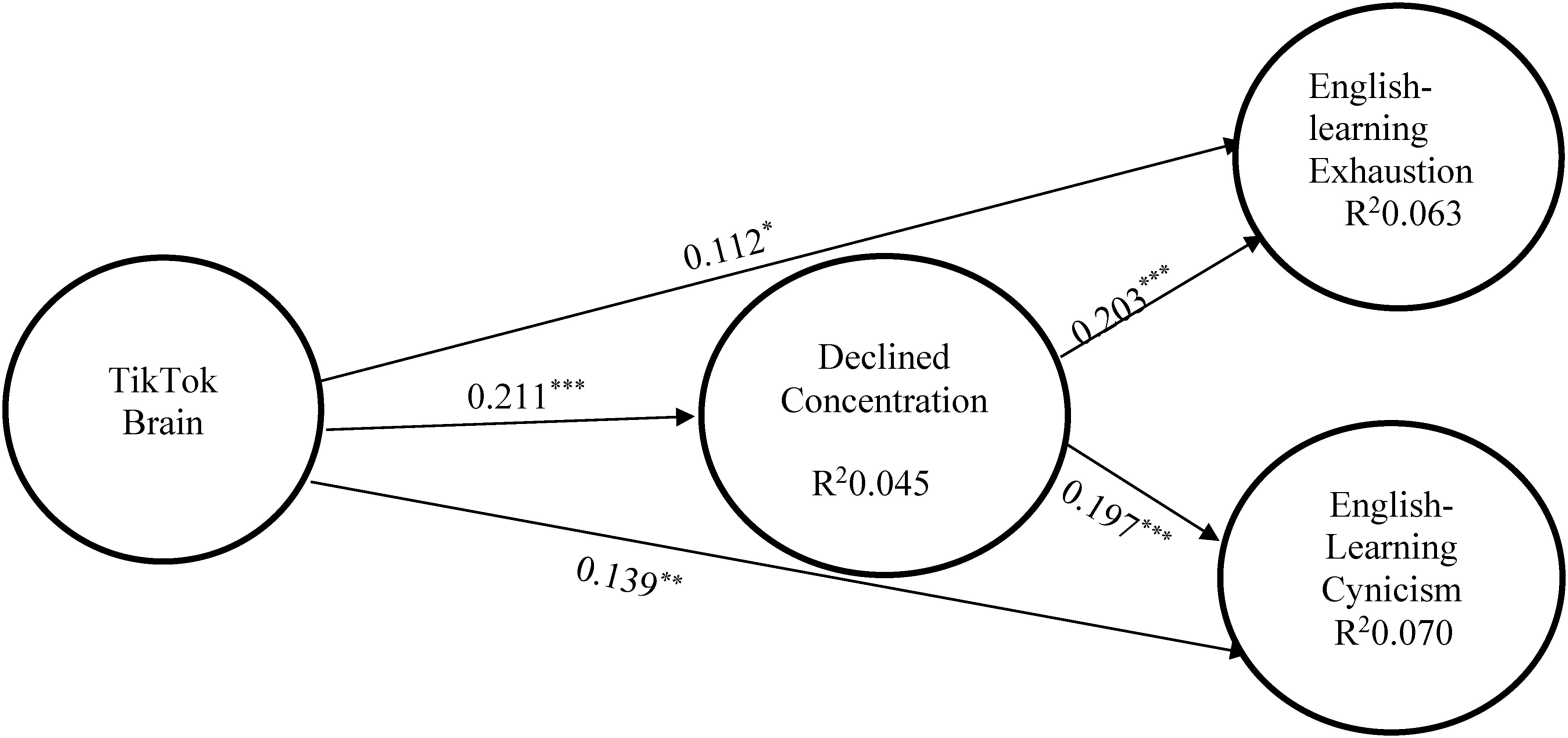
Structural model. ***p < 0.001, **p < 0.01, *p < 0.05.

Figure 2 displays the results of the structural model carried out by the Smart PLS. TB correlated with ELE (β = 0.112, *p* < 0.05) and ELC (β = 0.139, *p* < 0.01) positively, supporting H1 and H2. In addition, TB positively relates to DC (β = 0.211, *p* < 0.001) to support H3. Besides, DC showed positive associations with ELE (β = 0.203, *p* < 0.001) and ELC (β = 0.197, *p* < 0.001), supporting H4 and H5. Finally, for H6, the mediating path coefficient of TB − > DC − > ELE (β = 0.043, *t* value = 2.905, *p* < 0.01; LL = 0.019, UL = 0.076) and H7, TB − > DC − > ELC (β = 0.042, *t* value = 2.888, *p* < 0.01; LL = 0.018, UL = 0.074) is significant, supporting hypotheses H6 and H7.

## 5 Discussion

The descriptive statistics of this study indicate that the participants spent their daily time watching short videos ranging from 1 to 4 h, and this data distribution is consistent with the research results of Brunborg and Andreas (2019). Their study highlighted that teenagers spend an average of 2.5 h a day on social media, and approximately one-third of them spend 3 h on social media. The fact that these two research results support each other throughout the main interval increases the credibility of this study. Besides, this study agrees with Ye et al. (2023) that multi-dimensional data, including academic years and subject areas, were collected under self-determination theory, which made the collected data of university students more complete.

### 5.1 Direct relationships

First, the result showed that TikTok Brain favorably associated to declined concentration. The TikTok brain users have difficulty concentrating and are prone to distraction. This conclusion aligns with the study of Ye et al. (2025), who found that short-video addiction has a favorable effect on the TikTok brain and that TikTok brain users experience a decline in their capacity to control their own attention. Furthermore, Liu et al. (2023) confirmed that watching short videos for a long time would have concentrating problems. Therefore, the severity of TB is positively correlated with changes in brain activity patterns and the decline in the user’s attention control ability. This agrees with research by Tian et al. (2023), who discovered that overuse of short videos may relate to habitual distraction, making it difficult to focus on English learning.

Second, TikTok Brain is positively correlated with learning burnout, specifically, English-learning exhaustion and cynicism. Theoretically, this study offers support for that in the era of short videos, perceived pleasure does not increase students’ learning motivation; instead, it makes them more exhausted in learning. In addition, many students also regard watching short videos as an “escape outlet” to relieve study pressure and drive away fatigue (Liao, 2024). When learning English becomes dull and boring, or when they encounter insurmountable challenges, they will subconsciously turn on TikTok to seek that immediate relaxation and entertainment (Huang, 2025). Although this approach can bring comfort in the short term, in the long run, it will quietly strengthen a cognitive connection. Learning is painful, while entertainment is pleasant. Over time, students’ intrinsic motivation for learning English and their perseverance in persisting will gradually be weakened. In this way, a vicious circle will fall into the more tired one is from studying, the more one wants to have fun with short videos. The more addicted one is to short videos, the heavy academic burnout is, which is consistent with self-determination theory (Deci and Ryan, 2012).

Finally, declined concentration is positively correlated with learning burnout, aligning with the findings of Huang (2025). Foreign language learning requires maintaining a high level of concentration on reading, writing, listening, and speaking tasks (Yu et al., 2022; Wang et al., 2018). Lack of concentration will directly relate to difficulties in memorizing words, comprehension of texts, or omission of listening information. Such continuous inefficient efforts are likely to trigger a sense of frustration, which is a core component of learning burnout (Shi, 2024). A decline in attention and associated learning difficulties will gradually transform into emotional exhaustion (Tian et al., 2025), manifested as loss of interest, boredom, and even anxiety and depression (Maslach et al., 2001). Emotional exhaustion will also give rise to negative perceptions of English learning, such as believing that learning is meaningless, thereby weakening learning persistence, and getting the poor academic outcomes.

### 5.2 Mediate relationships

This study confirmed the mediating role of declined concentration. As Ye et al. (2025) have verified, students with the TikTok brain are addicted to short videos and have attention control problems. When people with attention control problems try to fight against temptation and complete tasks (Adrian et al., 2016), they often experience negative emotional states such as impatience and depression. Such emotional burden not only adversely contributes to psychological health, but also makes it difficult for people to focus on activities and hinders the fulfillment of assignments that need efforts of cognitive capacities and concentration (Rebetez et al., 2018). Koudsia and Kirchner (2024) also highlighted that cognitive overload can directly link to physical and mental fatigue and depression, ultimately predicting poor academic efficiency and achievements.

## 6 Conclusion

### 6.1 Implications

The results are based on Chinese EFL university students, which places the conclusions in a particular socio-educational context, but they have implications for a larger theoretical and practical discussion of the effects of digital media. First, this study constructed a theoretical framework based on the self-determination theory to understand the new phenomenon of learning burnout in the digital age. This is the first time that the cognitive neural mechanism of short-video consumption has been linked to foreign language learning burnout, revealing the cognitive impairments, and processing difficulties experienced by users who have the TikTok brain. This issue requires high attention and the formulation of relevant intervention strategies. Moreover, the results break through the limitations of traditional academic burnout research, which mostly focuses on academic pressure, lack of motivation, and teaching methods. It enriches the theoretical drivers of learning burnout and provides a key empirical direction for the long-term cognitive effects of digital media in the field of educational psychology.

At the practical level, this study provides direct inspiration for educational intervention, platform governance, and students’ self-regulation. For educators, it is necessary to redesign teaching strategies, such as integrating fragmented learning elements to adapt to students’ cognitive habits, while strengthening concentration training and deep learning guidance to alleviate burnout caused by cognitive conflicts. For short-video platforms, anti-addiction mechanisms and content diversity prompts should be set up to reduce their potential negative impact on the cognitive functions of teenagers. For students, research helps enhance their media literacy, making them aware that excessive consumption of short videos may undermine their learning endurance, and thus they can proactively manage their usage time. In addition, the research conclusions can promote universities to incorporate digital health and cognitive resource management into their student support systems and provide a scientific basis for formulating language teaching policies and mental health intervention plans for students in the digital age.

### 6.2 Limitations and further studies

The potential harmful consequences of frequent use of short-video platforms are revealed in this study. However, the relevant conclusions have the following limitations. Firstly, due to the cross-sectional design employed, the directionality of the associations between variables and the causal mechanisms cannot be ultimately determined. This requires further verification through longitudinal tracking or experimental research in the future. Secondly, in terms of sampling, although snowball sampling has improved the accessibility of specific groups, its non-probabilistic nature may, to some extent, weaken the representativeness of the sample for a broader student population, thereby affecting the external generalizability of the research conclusions. Future studies that adopt more random probability sampling methods will help enhance the diversity of the sample and the validity of the inferences. Finally, since the research samples were all drawn from EFL learners in Chinese universities, the generalization of the conclusions needs to be limited by considering specific contexts. Although there may be differences in specific effect values among different groups, the core mechanism revealed by this study still has theoretical implications that are applicable across different contexts and can provide a basis for subsequent path tests. Future research can be conducted in cross-cultural academic environments to verify the results, expand the sample age and language proficiency, and thereby improve and expand the conclusions of this study.

## Data Availability

The original contributions presented in this study are included in this article/Supplementary material, further inquiries can be directed to the corresponding author.
