# Supplementary material for: Academic burnout in the TikTok era: a battle between pleasure and concentration
Source: Front Psychol. 2026 Mar 5;17:1774030. doi: 10.3389/fpsyg.2026.1774030 (PMC12999888; doi:10.3389/fpsyg.2026.1774030)
Supplement: Supplementary file 1 [file Table_1.docx]

**Appendix A**

**TABLE A** Measurements

| **No.** | **TikTok Brian (Perceived Pleasure Enhancement)**  **抖音大脑 （感知愉悦）** |
| --- | --- |
| **I often need to watch TikTok short videos:**  **我经常观看短视频：** | |
| 1 | to make me feel happier.  来让我感到更快乐。 |
| 2 | to feel more energized.  来让自己更有活力。 |
| 3 | to enhance my mood.  来改善我的心情。 |
| 4 | to experience quick satisfaction.  来体验快速的满足感。 |
| 5 | to immerse myself in them.  来让自己沉浸其中。 |
| 6 | to feel joy and excitement.  来感受快乐和兴奋。 |
| 7 | to forget my troubles.  来忘记我的烦恼。 |
| 8 | to gain a sense of happiness.  来获得幸福感。 |
| 9 | to feel a heightened emotional state.  来感受更高的情绪状态。 |

| **No.** | **Declined Concentration**  **注意力下降** |
| --- | --- |
| **Since using short video apps,**  **自从我开始使用抖音短视频，** | |
| 10 | I find it hard to focus on one activity for a long time.  我发现很难长时间专注于一项活动。 |
| 11 | it often takes me some time to refocus my attention.  通常我需要一些时间来重新集中注意力。 |
| 12 | I find it difficult to calm myself down.  我发现很难让自己平静下来。 |
| 13 | I struggle to concentrate on my goals.  我很难集中精力去实现自己的目标。 |
| 14 | I often get distracted from what I am doing.  我经常会被一些事情分散注意力，无法专心做我正在做的事情。 |
| 15 | I find it hard to regain focus after being interrupted.  我发现自己在被打断之后很难重新集中注意力。 |

| **No.** | **English Learning Exhaustion**  **英语学习疲倦** |
| --- | --- |
| **English for me:**  **英语对我来说** | |
| 16 | I feel emotionally drained by my English studies.  我的英语学习让我感到精神上十分疲惫。 |
| 17 | I feel tired when I get up in the morning and I have to face English study another day at school. 早晨起床时，想到新的一天又 要面对英语，我会疲惫。 |
| 18 | Studying or attending an English class is really a strain for me.  学习或参加英语课程对我来说是一种折磨。 |
| 19 | I feel burned out from my English studies.  英语让我觉得自己被消耗殆尽。 |
| **No.** | **English Learning Cynicism**  **英语学习犬儒主义** |
| 20 | I have become less enthusiastic about my English studies.  我对英语学习的热情已经有所减退了。 |
| 21 | I have become increasingly indifferent to my English studies.  我对英语学习越来越冷漠。 |
| 22 | I’ve become more cynical about the potential usefulness of my English study.  我越来越怀疑学英语的用处。 |

**Appendix B**

**TABLE B** Common Method Variance (CMV)

| **Total Variance Explained** | | | | | | |
| --- | --- | --- | --- | --- | --- | --- |
| Component | Initial Eigenvalues | | | Extraction Sums of Squared Loading | | |
|  | Total | % of Variance | Cumulative % | Total | % of Variance | Cumulative % |
| 1 | 6.847 | 31.124 | 31.124 | 6.847 | 31.124 | 31.124 |
| 2 | 4.079 | 18.539 | 49.664 | 4.079 | 18.539 | 49.664 |
| 3 | 3.009 | 13.677 | 63.341 | 3.009 | 13.677 | 63.341 |
| 4 | 1.191 | 5.415 | 68.756 | 1.191 | 5.415 | 68.756 |
| 5 | .717 | 3.261 | 72.017 |  |  |  |
| 6 | .595 | 2.706 | 74.722 |  |  |  |
| 7 | .524 | 2.382 | 77.105 |  |  |  |
| 8 | .504 | 2.290 | 79.395 |  |  |  |
| 9 | .480 | 2.184 | 81.579 |  |  |  |
| 10 | .448 | 2.036 | 83.615 |  |  |  |
| 11 | .413 | 1.876 | 85.490 |  |  |  |
| 12 | .394 | 1.791 | 87.282 |  |  |  |
| 13 | .384 | 1.744 | 89.026 |  |  |  |
| 14 | .343 | 1.559 | 90.585 |  |  |  |
| 15 | .323 | 1.468 | 92.053 |  |  |  |
| 16 | .299 | 1.358 | 93.412 |  |  |  |
| 17 | .277 | 1.258 | 94.670 |  |  |  |
| 18 | .265 | 1.203 | 95.874 |  |  |  |
| 19 | .248 | 1.128 | 97.002 |  |  |  |
| 20 | .237 | 1.077 | 98.079 |  |  |  |
| 21 | .216 | .981 | 99.060 |  |  |  |
| 22 | .207 | .940 | 100.000 |  |  |  |

Extraction Method Principal Component Analysis
